# Prozone in malaria rapid diagnostics tests: how many cases are missed?

**DOI:** 10.1186/1475-2875-10-166

**Published:** 2011-06-15

**Authors:** Philippe Gillet, Annelies Scheirlinck, Jocelijn Stokx, Anja De Weggheleire, Hélder S Chaúque, Oreana DJV Canhanga, Benvindo T Tadeu, Carla DD Mosse, Armindo Tiago, Samuel Mabunda, Cathrien Bruggeman, Emmanuel Bottieau, Jan Jacobs

**Affiliations:** 1 Department of Clinical Sciences, Unit of Tropical Laboratory Medicine, Institute of Tropical Medicine (ITM), Nationalestraat 155, B 2000 Antwerp, Belgium; 2 Provincial Hospital of Tete (PHT), Mozambique; 3 Provincial Health Authorities, Tete, Mozambique; 4 National Malaria Programme, Maputo, Mozambique; 5 Department of Medical Microbiology, School for Public Health and Primary Care (CAPHRI), Maastricht University Medical Centre, Maastricht, The Netherlands

## Abstract

**Background:**

Prozone means false-negative or false-low results in antigen-antibody reactions, due to an excess of either antigen or antibody. The present study prospectively assessed its frequency for malaria rapid diagnostic tests (RDTs) and *Plasmodium falciparum *samples in an endemic field setting.

**Methods:**

From January to April 2010, blood samples with *P. falciparum *high parasitaemia (≥ 4% red blood cells infected) were obtained from patients presenting at the Provincial Hospital of Tete (Mozambique). Samples were tested undiluted and 10-fold diluted in saline with a panel of RDTs and results were scored for line intensity (no line visible, faint, weak, medium and strong). Prozone was defined as a sample which showed no visible test line or a faint or weak test line when tested undiluted, and a visible test line of higher intensity when tested 10-fold diluted, as observed by two blinded observers and upon duplicate testing.

**Results:**

A total of 873/7,543 (11.6%) samples showed *P. falciparum*, 92 (10.5%) had high parasitaemia and 76 were available for prozone testing. None of the two Pf-pLDH RDTs, but all six HRP-2 RDTs showed prozone, at frequencies between 6.7% and 38.2%. Negative and faint HRP-2 lines accounted for four (3.8%) and 15 (14.4%) of the 104 prozone results in two RDT brands. For the most affected brand, the proportions of prozone with no visible or faint HRP-2 lines were 10.9% (CI: 5.34-19.08), 1.2% (CI: 0.55-2.10) and 0.1% (CI: 0.06-0.24) among samples with high parasitaemia, all positive samples and all submitted samples respectively. Prozone occurred mainly, but not exclusively, among young children.

**Conclusion:**

Prozone occurs at different frequency and intensity in HRP-2 RDTs and may decrease diagnostic accuracy in the most affected RDTs.

## Background

Currently malaria rapid diagnostic tests (RDTs) detect *Plasmodium *antigens in blood by antibody-antigen interactions on a nitrocellulose test strip. The targeted antigens include those specific to *Plasmodium falciparum *(histidine-rich protein-2 (HRP-2) and *P. falciparum*-specific parasite lactate dehydrogenase (Pf-pLDH)) and antigens common to *P. falciparum, Plasmodium vivax*, *Plasmodium ovale *and *Plasmodium malariae *(pan-species pLDH and aldolase). RDTs combine a control line with one, two or three antigen-detecting test lines, and are referred to as two-, three- and four-band RDTs respectively.

RDTs are being rolled out as an alternative to microscopic diagnosis in malaria endemic settings [1] and have demonstrated sensitivities close to 100% for the detection of *P. falciparum *at densities above 100 asexual parasites/μl or > 0.002% of parasitized red blood cells (RBC). Most false-negative results occur at lower parasite densities. However, false-negative results have been reported also at high parasite densities. Part of those may be ascribed to genetic variations of the HRP-2 [2-6], but the prozone phenomenon may also be involved. Prozone is defined as false-negative or false-low results in antigen-antibody immunological reactions, due to an excess of either antigens or antibodies [7,8]. In RDTs, the prozone has been observed in samples with high *P. falciparum *parasite densities and dilution of the sample can trace and correct the effect [9]. In a recent laboratory evaluation, RDT brands were challenged to a panel of clinical samples with *P. falciparum*. Prozone was observed among 16 out of 17 HRP-2 RDTs but not among five Pf-pLDH RDTs [9]. However, as this was a retrospective laboratory study in a reference setting, the frequency and impact of the prozone effect on the diagnosis of malaria in the daily practice of endemic settings remains to be determined.

The main aim of the present study was to assess the frequency of the prozone effect in a malaria endemic field setting. A subsidiary aim was the confirmation of the previous observation that HRP-2 RDTs, but not Pf-pLDH tests, are affected by prozone [9].

## Methods

### Study site, study period and patients included

The study was conducted in the emergency ward of the Provincial Hospital of Tete (PHT), located in Central Mozambique. In this area, malaria is predominantly caused by *P. falciparum*. Transmission is perennial with peaks during and at the end of the rainy season (February - April)[10,11].

The PHT serves as a reference hospital for Tete Province (1,700,000 inhabitants). According to hospital statistics, yearly approximately 50.000 patients present themselves with clinical suspicion of malaria. Diagnosis is confirmed in about 20% of them (either by RDT or microscopy). For this study, all patients suspected of malaria and presenting at the emergency ward of the PHT were prospectively included on a 24 hours/7 days basis from January till April 2010.

### Patients, samples and diagnostic work-up

Routine procedures for malaria diagnosis (following national guidelines) at PHT are as follows: EDTA-anticoagulated blood is sampled and a full blood count is performed by an automated haematology analyzer (KX-21N, Sysmex, Kobe, Japan). For children ≤ 5 years, malaria diagnosis is made on a thick blood film (TBF). For patients above five years of age, an RDT is performed and in case of a positive result a TBF is made for confirmation and determination of parasite density. TBFs are stained for 20 minutes at pH 7.2 using Giemsa 3.5% (Merck, KGmA, Darmstadt, Germany). According to the national malaria clinical guidelines, parasite density is scored on a semi-quantitative scale from 1+ (1-9 asexual parasites/100 high power microscopic fields) to 5+ (> 100 asexual parasites/1 high power microscopic field) [12]. During the study period, three RDT brands (*ICT Malaria, Paracheck-Pf and SD Malaria Antigen Pf FK50*) were routinely used (provided by the national malaria programme or a partner NGO).

For the purpose of the study, demographic data, presenting symptoms and clinical signs of severe malaria [13] were recorded for all patients with clinical suspicion of malaria attending the PHT. In addition, TBFs were performed for all suspected patients, irrespective of their age. For TBFs positive for *P. falciparum *and scored as 4+ or 5+, parasite densities were quantitatively assessed: the number of asexual parasites was counted against 200 white blood cells (WBC) and converted to parasites/μl using the WBC count/μl. Hereafter, values were converted to % of parasitized RBC using the RBC count/μl. WBC and RBC counts were those provided by the haematology analyzer.

Samples with a high parasitaemia, defined as ≥ 4% of parasitized RBC [14,15], were challenged against a panel of RDTs consisting of four HRP-2 RDTs and two Pf-pLDH RDTs (see below and Table 1). Determination of parasite density and testing of RDTs were done at the latest 48 hours after sampling and samples were stored at 4°C pending RDT testing. Analyses were performed by the regular laboratory staff as well as the authors PG, AS, JS and HC. Left-overs of the EDTA blood samples were stored at -20°C till the end of the study for further analyses.

**Table 1:** Panel of RDT brands used in the study

Brands/manufacturers and Lot numbers	Format and *Plasmodium *antigens targeted (*P. falciparum *target is underlined)	WHO FIND Procurement list *	WHO FIND Evaluation ^†^
*ICT Malaria *ICT Diagnostics, Cape Town, South Africa Lot n°: 32784	Two-band: HRP-2	yes	yes
*Paracheck-Pf *Orchid Biomedical Systems, Goa, India Lot n°: A31003, 31672, 32972, 31797	Two-band: HRP-2	yes	yes
*SD Malaria Antigen Pf FK50 *Standard Diagnostics, Hagal-Dong, Korea Lot n°: 082011, 082012, 080216	Two-band: HRP-2	yes	yes
*SD Malaria Ag Pf/Pan FK60 *Standard Diagnostics, Hagal-Dong, Korea Lot n°: 090008, 090010, 090026	Three-band: HRP-2, pan-pLDH	yes	yes
*Hexagon Malaria Combi *^‡ ^Human Wiesbaden, Germany Lot n°: 80930	Three-band: HRP-2, aldolase	no	yes
*Malaria Pan/Pv/Pf Rapid Device*^‡ ^Biotec laboratories Ltd., Ipswitch, UK Lot n°: 91081, 91100	Four-band: HRP-2, Pan-pLDH, Pv-pLDH	no	no

*CareStart Malaria pLDH *Acces Bio, New Jersey, USA Lot n°: B191L, A101L	Three-band: Pf-pLDH, pan-pLDH	no	yes
*SD Malaria pLDH FK40 *Standard Diagnostics, Hagal-Dong, Korea Lot n°: 081006	Three-band: Pf-pLDH, pan-pLDH	no	yes

*First Response Malaria Ag Combo *^$^ Premier Medical Coorporation Ltd., Daman, India Lot n°: 6900309	Three-band: HRP-2, Pan-pLDH	yes	yes
*Hexagon Malaria *^$^ Human, Wiesbaden, Germany Lot n°: 0001	Two-band:: HRP-2	no	yes
*ICT Malaria Combo *^$^ ICT Diagnostics, Cape Town, South Africa Lot n°: 32250	Three-band: HRP-2, Aldolase	no	yes
*Malaria Total Quick *^$^ Cypress Diagnostics, Leuven, Belgium Lot n°: 090002	Three-band:HRP-2, Pan-PLDH	no	no

* Included in the interim selection for procurement of malaria rapid diagnostic test [43].

^† ^Evaluated by WHO/FIND [16,17].

^‡ ^RDTs assessed for prozone on stored samples at the end of the study.

^$ ^Additional panel of RDTs assessed at the end of the study with a subset of stored samples positive for prozone for at least one of the prospectively assessed HRP-2 RDTs.

### Malaria rapid diagnostic tests used and test procedures

Malaria RDTs in cassette format were selected based on demonstrated diagnostic accuracy [16-18], use by national malaria control programmes and non-governmental organizations (NGOs) and availability in Mozambique.

For each sample with high parasitaemia, a 10-fold dilution was made in saline. Both undiluted and diluted sample were assessed with each RDT brand and tests were run in duplicate and read by two observers. The second observer was blinded to the first observer's readings. Tests were performed according to the instructions of the manufacturer, except for the use of an automatic pipette (Finnpipette, Helsinki, Finland) instead of the RDT kits' transfer device. Test line intensities were scored into five categories: none (no line visible), faint (barely visible), weak (paler than the control line), medium (equal to the control line) and strong (stronger than the control line) [19]. When a control line did not appear, the test was interpreted as invalid and the sample was retested. To assure timely readings, tests were performed in time-controlled batches. Readings were carried out at daylight, within the prescribed reading delay.

### Additional analyses

Two additional HRP-2 RDT brands (*Hexagon Malaria Combi *and *Malaria Pan/Pv/Pf Rapid device*), not delivered in time for the prospective analysis, were assessed at the end of the study period with samples that had been stored at -20°C (maximum storage duration: 121 days).

In order to investigate the prozone occurrence at parasite densities below 4%, all RDT brands were also assessed with a subset of 45 samples scored as 4+ or 5+ but with parasite densities below 4%.

To confirm the prozone susceptibility of HRP-2 RDTs, an additional panel of four HRP-2 based RDTs was assessed with a subset of stored samples that had demonstrated prozone for at least one of the prospectively assessed HRP-2 RDTs (Table 1). Among the evaluated RDTs, there was also an additional lot number of *Malaria Pan/Pv/Pf Rapid Device*. The results of these latter panels were not included for calculation of the frequency of prozone.

### Test outcomes and definitions

Samples with high parasitaemia were defined as samples with parasite densities ≥ 4%. Prozone was defined if a sample showed no visible test line or a faint or weak test line when tested undiluted, and a visible test line of higher intensity when tested 10-fold diluted, as observed by two blinded observers and upon duplicate testing [9].

The frequency of prozone was extrapolated against the total number of samples with high parasitaemia, the total number of *P. falciparum *positive samples and the total number of patients suspected of malaria.

### Quality control

At the start of the study, the laboratory staff received a refresher course on malaria microscopy and RDTs. The study team was trained on study procedures and flow during a pilot phase.

All RDTs were purchased in Belgium and shipped to Tete, except for *SD malaria Antigen Pf FK 50 *and the lot A31003 of the *Paracheck-Pf *which were provided locally. During shipment and storage, temperature and humidity were monitored using loggers (Ebro Electronic GmBH, Ingolstadt, Germany). On a daily basis, 10% of TBFs were randomly elected and reread by a member of the study team who was blinded to the original result. All discordances were resolved by a third reader's reading. A photograph was taken from all RDT tests performed and the TBFs were stored for quality control.

### Data management and statistical analysis

According to the initial sample size calculation, a total of 5,700 patients suspected of malaria were required for reliable estimation of the frequency of prozone. This was based on the following assumptions and information from hospital statistics: malaria-attributable fraction during the wet season of 30%, prevalence of hyperparasitaemia of 10%, prozone frequency among hyperparasitaemia samples of 30% with 95% confidence intervals (CI) between 20% and 40%.

Data were recorded in registers and on individual case report forms. Data were entered in Microsoft Access (Microsoft Corporation, Redmond, Washington USA) and analyzed in Stata 11.1 (StataCorp LP, College Station, USA). Differences between proportions were tested for significance using the Pearson's Chi-square test or, in case of small sample sizes, a two-tailed Fisher's exact test. Reproducibility and inter-observer reliability for line intensity readings were assessed using the kappa statistic for paired observers and percentage agreements. Differences between medians were tested using the Wilcoxon test. Lot variations for matched pairs of samples were assessed using the McNemar's test. The relation between line intensities of prozone samples across the parasite densities was assessed using the Cuzick's test for trend. A p-value < 0.05 was considered as significant.

### Ethical review

The study was approved by the Institutional Review Board of ITM, by the ethical committee of Antwerp University, Belgium and by the Comité Nacional de Bioética para a Saude (MoH), Mozambique. Patients, children's parents or guardians were informed in Portuguese or in the local language (Nhungue) and their written consent was required prior to enrolment.

## Results

### Patients, samples included and flow chart of the study

**Figure 1:**
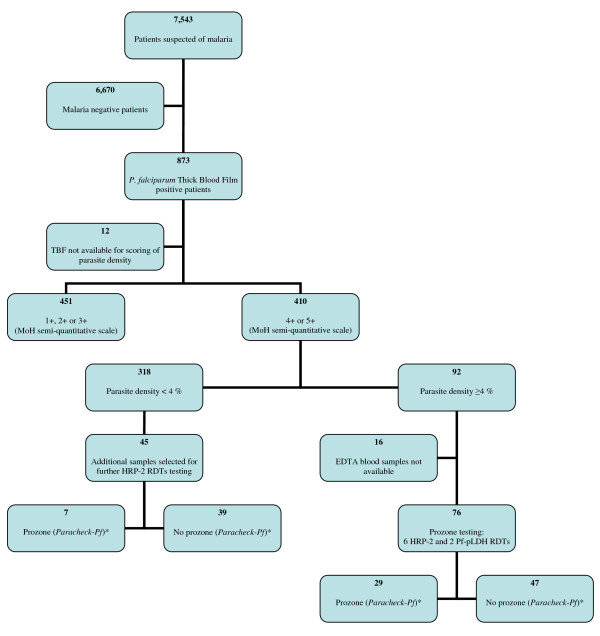
**Flow of patients and samples**. Footnotes figure 1: * Prozone results presented for *Paracheck-Pf*, results for other RDTs are listed in Table 3. For definition of prozone see text.

During the study period, a total of 7,543 patients with suspicion of malaria were included, of whom 873 (11.6%) were diagnosed with *P. falciparum *infection by thick blood film (Figure 1). About half of the positive patients with available TBF had parasite densities of 4+ or 5+ (410/861, 47.6%) and 92 had parasite densities ≥ 4%. Table 2 summarizes the demographic and parasitological characteristics of malaria positive patients with complete available data.

**Table 2:** Characteristics of *P. falciparum *samples included

Parasite density (MoH semi-quantitative scale)	Age group (nrs)				
	
	Children 0 - 5 years	Children 5-14 years	Adults (≥ 15 years)	No data	Total
1+	39	19	97	12	167
2+	24	18	85	11	138
3+	27	20	91	8	146
4+	81	40	110	9	240
5+	97	32	36	5	170

Total	268	129	419	45	861

### Quality assessment, invalid test results, reproducibility and inter-observer reliability

Shipment and storage temperatures of RDTs ranged between 4.0°C and 23.5°C (mean 17.5 ± 8.7°C) and 23.3°C and 32.3°C (mean 28.9 ± 1.5°C) respectively. Storage temperatures exceeded the highest allowed temperature (30°C) for three RDT brands *Hexagon Malaria Combi, Malaria Pan/Pv/Pf Rapid Device and CareStart Malaria pLDH*): temperatures of 31°C and 32°C were registered for a cumulative period of 7.6 and 1 days respectively. Invalid results were observed for five brands and ranged between 0.2% and 0.7% of tests performed; upon repetition all tests performed well. For the HRP-2 line intensity readings, the overall agreement among the two observers ranged from 86.1% (*Paracheck-Pf*) to 97.1% (*SD malaria Ag Pf/Pan FK 60*) and kappa values ranged from 0.67 (*Malaria Pan/Pv/Pf rapid devi*ce) to 0.90 (*ICT Malaria*). The reproducibility of the RDTs among the duplicate tests in terms of HRP-2 line intensity ranged from 71.7% (*Hexagon Malaria Combi*) to 97.4% (*SD malaria Ag Pf/Pan FK 60*) and kappa values ranged from 0.5 (*Hexagon Malaria Combi*) to 0.8 (*SD malaria Ag Pf/Pan FK 60*).

### Frequency of the occurrence of prozone

**Figure 2:**
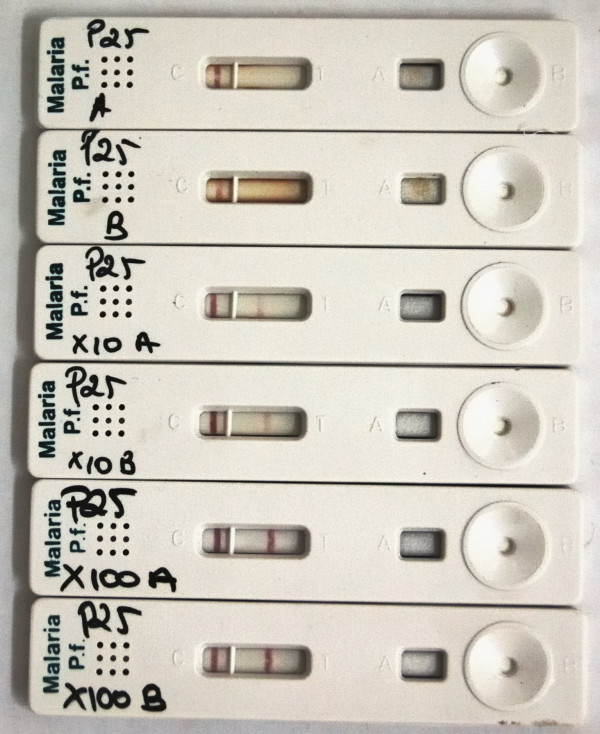
**Example of prozone for *Paracheck-Pf***. Footnotes figure 2**: ***Paracheck-Pf *RDT cassettes run with a blood sample infected with *P. falciparum *at a parasite density of 8.3%. The sample was assessed in duplicate, undiluted (P25 A and P25 B), 10 × diluted (P25 × 10A and P25 × 10B) and 100 × diluted (P25 × 100A and P25 × 100B). All cassettes show regular control lines, cassettes P25 A and B show no visible test line. Cassettes P25 × 10A and P25 × 10B show weak test lines and the maximum line intensity (strong) was obtained after 100 × dilution (cassettes P25 × 100A and P25 × 100B).

Prozone affected all six HRP-2 RDT brands in proportions ranging from 6.7% to 38.2% of samples tested. The two Pf-pLDH RDTs did not show any prozone positive sample. Table 3 lists for each RDT brand and samples with high parasitaemia the frequencies for which prozone was observed, matched with the line intensity of the undiluted sample. Among 104 test results with prozone, negative, faint and weak HRP-2 test lines were observed in 4 (3.8%), 15 (14.4%) and 85 (81.7%) results. Two RDT brands accounted for all negative and faint test lines. The three samples with negative test lines (including one sample with negative results for two RDT brands) had parasite densities of 8.3%, 8.3% and 8.4% (Figure 2).

**Table 3:** Number of *P. falciparum *samples with parasite density ≥ 4% generating prozone for HRP-2 (n = 6) and Pf- pLDH (n = 2) RDT brands.

Brands/manufacturers	*P. falciparum *antigen targeted	Number of samples tested	Total number of samples with prozone (%)	HRP-2 or Pf-pLDH line intensity for undiluted prozone positive samples		
				
				Negative	Faint	Weak
*Paracheck-Pf*	HRP-2	76	29 (38.2)	3	5	21
*ICT Malaria*	HRP-2	76	27 (35.5)	1	10	16
*SD Malaria Antigen Pf FK50*	HRP-2	76	25 (32.9)	-	-	25
*Hexagon Malaria Combi*	HRP-2	72	12 (16.7)	-	-	12
*SD Malaria Ag Pf/Pan FK60*	HRP-2	76	6 (7.9)	-	-	6
*Malaria Pan/Pv/Pf Rapid Device*	HRP-2	75	5 (6.7)	-		5

*SD Malaria pLDH FK40*	Pf-pLDH	76	-	-	-	-
*CareStart Malaria pLDH*	Pf-pLDH	76	-	-	-	-

Table 4 gives a breakdown of the proportions of prozone for the most affected (in terms of line intensities) RDT brand (*Paracheck-Pf*) according to different denominators. As an example, the proportions of false negative or faint HRP-2 lines were 10.9%, 1.2% and 0.1% among the samples with parasite densities ≥ 4%, all *P. falciparum *positive samples and all suspected samples respectively. For the *ICT malaria *similar proportions were 14.1% (CI: 7.74-22.95); 1.5% (CI: 0.80-2.53) and 0.2% (CI: 0.09-0.29) respectively.

**Table 4:** Proportion (%) of prozone for *Paracheck-Pf *according to different denominators, 95% binomial confidence intervals (CI) within brackets

Category	Number	% of patients suspected of malaria	% of *P. falciparum *positive patients	% of samples with high parasitaemia
Patients suspected of malaria (1 sample per patient included)	7,543	100		
Samples positive for *P. falciparum*	873	11.6 (10.86-12.32)	100	
Samples scored as 4+ or 5 + (MoH semi-quantitative scale)	410	5.4 (4.93-5.97)	47.0 (43.61-50.34)	
Samples with a high parasitaemia (parasite density ≥ 4%)	92	1.2 (0.98-14.9)	10,5 (8,58-12,77)	100
Number of samples available for prozone testing	76	-	-	-
Negative, faint or weak HRP-2 test lines *	29	0.5 ^†^ (0.32-0.64)	4.0 ^†^ (2.81-5.53)	38.0 ^†^ (28.12-48.76)
Negative or faint HRP-2 test lines *	8	0.1 ^†^ (0.06-0.24)	1.2 ^†^ (0.55-2.10)	10.9 ^†^ (5.34-19.08)
Negative HRP-2 test lines *	3	0.05 ^†^ (0.01-0.14)	0.5 ^†^ (0.12-1.17)	4.4 ^†^ (1.20-10.76)

* Line intensity for undiluted samples

^† ^Proportions calculated according to Table 2, corrected for the number of samples that were not available for prozone testing (n = 16).

Table 5 lists the clinical and laboratory data for the samples with and without prozone for *Paracheck-Pf*. Among the high parasitaemia samples, prozone occurred most frequently but not exclusively among the group less than five years of age. No significant relation between prozone and the presenting symptoms was found. However, cough was significantly more prevalent among the prozone positive group. Of note, clinical signs of severity were not associated with prozone, and, moreover, prozone occurred also in 10 (36%) patients without any sign of severe malaria. Among the laboratory values, the prozone group was significantly associated with lower hemoglobin levels and RBC counts. Parasite densities of prozone positive and prozone negative samples did not differ significantly.

**Table 5:** Characteristics of *P. falciparum *samples that were prozone positive or negative for *Paracheck-Pf*

		Samples with high parasitaemia (parasite density ≥ 4%)			
		
		Prozone positive	Prozone negative	Total	p = *
		**n = 29**	**n = 47**	**n = 76**	
**Demographic and clinical data (expressed as % of total)**					
Patients (n= 76)	Children < 5 years (n = 53)	82.8	61.7	69.8	NS
	Children ≥ 5 years (n = 14)	10.3	23.4	18.4	NS
	Adults (> 14 years) (n = 9)	6.9	14.9	11.8	

Gender ratio (n = 74)	Male/female	40.7	57.4	51.4	NS

Presenting symptoms	Fever (n = 64)	96.3	97.3	96.9	NS
	Cough (n = 59)	50.0	11.4	27.1	0.002
	Vomiting (n = 60)	28.0	45.7	38.3	NS
	Diarrhoea (n = 60)	28.0	45.7	30.0	NS

Clinical signs of severity	At least one (n = 60)	64.0	48.6	55.0	NS

Laboratory sign of severity	Haemoglobin < 5 g/dl (n = 76)	20.7	6.4	11.8	NS

Signs of severity	At least one clinical or laboratory (n = 61)	69.2	54.3	60.7	NS

**Laboratory values (expressed as median and 95% CI)**					

Parasite density in % (n = 76)	Median	8.0	6.9	6.9	NS

	Range	4.0-28.7	4.2-22.3	4.0-28.7	

Hemoglobin level (g/dl) (n = 76)	Median	7.2	9.0	8.3	0.0074

	Range	2.9-12.5	2.5-13.3	2.5-13.3	

RBC count (× 10^9^/l) (n = 76)	Median	3.1	3.8	3.4	0.0131

	Range	1.1-5.3	0.9-5.3	0.9-5.3	

Platelet count (× 10^6^/l) (n = 76)	Median	74	90	86	NS

	Range	12-488	13-390	12-390	

WBC count (× 10^6^/l) (n = 76)	Median	10.4	10.1	10.2	NS

	Range	4.4-19.9	3.2-27.6	3.2-27.6	

* NS = not significant

Table 6 lists the number of HRP-2 based brands affected by prozone in relation to parasite density as well as the results for the subset of 45 additional samples of parasite densities < 4% (mean ± SD parasite density: 2.7 ± 0.9%). Prozone occurred less frequently in samples with parasite densities < 4% compared to samples with high parasitaemia (13/45 versus 39/76, p = 0.016). Prozone occurred dispersedly among the samples but the intensity of prozone (in terms of numbers of negative, faint or weak test lines among undiluted samples) increased with parasite density (p = 0.015). The three samples with prozone in five out of six HRP-2 RDTs had parasite densities of 8.7%, 11.5% and 28.7% respectively. Among the samples with parasite densities < 4%, samples with negative test lines were not observed and prozone with faint test lines was observed for only one brand (*ICT Malaria*, at parasite densities of 2.2%, 2.9% and 3.4% respectively).

**Table 6:** Number of HRP-2 RDT brands affected by prozone in relation to the parasite density and HRP-2 test line intensity for undiluted samples (negative, faint and weak)

Parasite density %	Number of samples assessed	Prozone for at least 1RDT		Prozone for at least 2 RDTs		Prozone for at least 3 RDTs		Prozone for at least 4 RDTs		Prozone for at least 5 RDTs	
		
		Weak	Faint or no line	Weak	Faint or no line	Weak	Faint or no line	Weak	Faint or no line	Weak	Faint or no line
1 to 3.9	45	10	3 *	8	-	5	-	2	-	-	-
4 to 4.9	18	4	4 ^†^	8	0	7	-	2	-	-	-
5 to 9.9	39	13	8 ^‡^	11	2 ^£^	8	-	4	-	1	-
≥ 10	19	6	4 ^$ ^	8	1 ^+^	8	-	3	-	2	-

* Parasite densities of 2.2, 2.9 and 3.4%

^† ^Parasite densities of 4.1, 4.2, 4.3 and 4.7%

^‡ ^Parasite densities of 8.0, 8.0, 8.3, 8.3, 8.4, 8.6, 8.9 and 9.3%

^£ ^Parasite densities of 8.0 and 8.3%

^$ ^Parasite densities of 10.6, 11.0, 11.5 and 28.7%

^+ ^Parasite densities of 28.7%

Table 7 lists the results for the additional HRP-2 RDT brands assessed with samples that were prozone positive for at least one of the brands assessed prospectively. These results confirmed the susceptibility of the HRP-2 RDT brands to prozone: prozone affected all four brands in proportions ranging from 20.6% to 85.0%. Although no negative test results were obtained, faint test lines were observed in proportions up to 17.5%. In addition, there was a clear difference between the two lot numbers of the *Malaria Pan/Pv/Pf Rapid Device*: for 43 samples assessed by both lots, prozone was observed in 5 (11.6%) versus 22 (51.2%) samples respectively (p < 0.0005).

**Table 7:** Number of prozone positive samples for additional HRP-2 RDT brands that were assessed with samples positive for prozone with at least one of the RDTs from Table 3

Brands/Manufacturers	*P. falciparum *antigen target	Number of samples tested	Total number of samples with prozone (%)	HRP-2 or Pf-pLDH line intensity for undiluted prozone positive samples		
				
				Negative	Faint	Weak
*Hexagon Malaria*	HRP-2	39	13 (33.3%)	-	0	13
*Malaria Total Quick*	HRP-2	44	22 (50.0%)	-	5	17
*First Response Malaria Ag Combo*	HRP-2	40	34 (85.0%)	-	7	27
*ICT Malaria Combo*	HRP-2	34	7 (20.6%)	-	1	6
*Malaria Pan/Pv/Pf Rapid*^‡ ^*Device*	HRP-2	43	22 (51.2%)	-	4	18

## Discussion

In 2009, an estimated 225 million cases of malaria occurred with 781.000 deaths, mostly due to *P. falciparum *in children in Africa [1]. WHO recommends parasitological diagnostic testing before treatment. When microscopy is not available, RDTs are the alternative. RDTs have been demonstrated to perform equally well or even better than microscopy in field settings [20-23] and are currently deployed at all levels of health facilities [1].

Some limitations are however to be mentioned. The wet season of 2009-2010 in Mozambique was characterized by irregular rainfall and long dry spells. Due to this low rainfall (30% below expected value in November 2009 up to 60% in March 2010 [24]) and the impact of local control measures in the months preceding the study, there was less malaria than expected and the originally planned 5% parasite density threshold (defined by WHO as hyperparasitaemia [13]) was replaced by 4%, also used in other studies [14,15]. Although samples below this 4% threshold were included, prozone was not assessed below this threshold, precluding systematic study of possible clinical or laboratory predictors of prozone. Further, due to non-availability of trained staff around the clock, it was not always possible to record all clinical data and to work-up all samples in the laboratory. Likewise, for logistic reasons, two RDTs were assessed with blood samples stored at -20°C and not on fresh samples. However, it should be noted that the HRP-2 antigen is very stable and resistant to harsh conditions [25]. For three RDTs, storage temperatures slightly exceeded those recommended by the manufacturers. Finally only a minority of undiluted prozone positive samples showed no visible test line, whereas the remaining samples showed faint or weak test lines. However, disregarding faint or even weak test lines as negative is a common error among end-users in field settings [26-28].

There are only few original studies reporting on prozone in malaria RDTs [9,29]. Prozone may however explain for the rare but consistent reports of false negative HRP-2 based RDT results in samples with high parasite density: for instance, in a recent study of two RDTs in Sierra Leone, two false-negative samples were observed with the HRP-2 RDT but not with the Pf-pLDH RDT. The parasite densities of both samples were 288,000/μl and 580.000/μl, corresponding to 5.7% and 11.6% parasite density respectively [30]. Interestingly, the HRP-2 test used in this study was *Paracheck-Pf *and the two samples accounted for 1.1% of all malaria positive samples which is in line with the present findings. Similar observations affecting *Paracheck-Pf *or other HRP-2 RDT brands have been reported from endemic as well as non-endemic settings [31-33].

In line with previous findings, the present study demonstrated prozone in HRP-2 but not in Pf-pLDH based RDTs frequently used in field settings [9]. Compared to HRP-2 based RDTs, pLDH based RDTs are ascribed lower sensitivity and lower heat-stability [23,34,35], but according to a recent field study and the second WHO/FIND RDT evaluation round, Pf-pLDH RDTs may perform equally well as HRP-2 RDTs [17,36]. The observation of lot-to-lot variations in one HRP-2 RDT brand illustrates that small differences in composition may influence the vulnerability to prozone. In that way, it should be noted that the present data only reflect those of the examined lot numbers and may therefore not be extrapolated to all RDT brands. Although not assessed in the present study, several factors in the design of the individual RDT brands may explain for their different vulnerabilities to the prozone effect. As antigen-antibody interactions are time-related, factors influencing the speed of migration may be involved, such as the pore size of the nitrocellulose membrane and the viscosity and volume of the buffer. In addition, the structure of the antibodies, the affinity and the avidity of the antigen-antibody binding may be of influence. With regard to end-user practice, application of a high blood volume may increase the risk of prozone, as demonstrated previously [9].

From the present results, it is clear that prozone occurred dispersedly among samples with high parasitaemia. Prozone with non-visible test lines occurred exclusively in samples with parasite densities above 8% and the frequency and intensity of prozone decreased below the 4% threshold. However, the association parasite density - presence of prozone among samples with parasite densities ≥ 4% was not straightforward. This may be ascribed to factors such as capillary sequestration of the parasites, variations in the antigen production during the cycle and strain differences [4,37]. Prozone remains a rare phenomenon and although the present study was not designed to trace risk factors of prozone, this study suggests that, apart from hemoglobin level, there are no clear indicators for prozone. This relation can be explained by the high concentration of HRP-2 related to the duration of the disease and/or to the number of malaria crises in the past few weeks.

For the two most affected RDT brand (*Paracheck-Pf and ICT Malaria*), prozone with negative or faint HRP-2 test lines (the most dangerous situation) occurred in at least 1.2% of malaria-positive samples and 10.9% of samples with high parasitaemia. At such frequencies, the diagnostic accuracy may be affected and the impact on predictive values will depend on the malaria-attributable fraction of fevers and the proportion of high parasite densities: both factors are related to transmission intensity and pre-existing immunity of the affected population [38]. For Africa, the *P. falciparum *prevalence rate in children aged two to ten years is actually estimated at 17% [39]. For the distribution of parasite densities, published data are scarce. Two recent studies conducted in children in areas of perennial transmission in Gabon and Sierra Leone reported median parasite densities of 13,860/μl (1,400 - 71,452) and 264,000/μl (1 - 2,136,000) [22,30]. When extrapolating for Mozambique, based on the 4,310,086 suspected malaria cases reported in 2009 [1], a 17% malaria attributable fraction [39] and a 10% proportion of high parasitaemia, the annual numbers of negative or faint test lines were calculated as 7,694 (CI: 3,883-13,922) and 9,643 (CI: 4,543-15,387) with *Paracheck-Pf *and *ICT Malaria *(tested with the presently evaluated lots) respectively.

The risk related to false-negative RDT results due to low parasite densities is mitigated by diagnostic algorithms recommending to repeat testing after an unexpected negative RDT result [40-42]. However, such policy will not timely correct for false-negative results due to the prozone, as hyperparasitaemia represents a life-threatening situation. In addition, there is a tendency to roll out RDTs to poorly resourced peripheral health care facilities where there are no further laboratory facilities to perform sample dilution or microscopy in order to correct for prozone [1,20]. Possible other measures to address prozone are training of the end-user to understand the problem and to assure interpretation of faint test lines as positive test results. Concerning RDT quality control at the level of national reference laboratories and the FIND/WHO lot testing programme, samples with hyperparasitaemia could be included but in view of the low prozone frequency and its scattered distribution among samples with hyperparasitaemia, it is difficult to assess prozone on a pre-release basis. Post-marketing follow-up including incident reporting could provide further clues. Finally, depending on the distribution of parasite densities in a given population, susceptibility to prozone should be added as a major argument in the strategic choice between Pf-pLDH and HRP-2 RDTs.

In conclusion, prozone is a rare event but it occurs among widely used HRP-2 RDTs at frequencies that may diminish diagnostic accuracy of the affected RDTs.

## List of abbreviations

Ag: Antigen; CI: confidence intervals; EDTA: Ethylene Diamine Tetra-acetic Acid; FIND: Foundation for Innovative New Diagnostics; HRP-2: histidine-rich protein-2; ITM: Institute of Tropical Medicine; NaCl: Sodium Chloride; MoH: Ministry of Health; *P*.: Plasmodium; *Pf*: Plasmodium falciparum; PHT**: **Provincial Hospital of Tete; pan-pLDH: pan species parasite lactate dehydrogenase; Pf-pLDH: *Plasmodium falciparum*-specific parasite lactate dehydrogenase; pLDH: parasite lactate dehydrogenase; RDT(s): Rapid Diagnostic Test(s); TBF(s): thick blood film(s); WBC: white blood cell; RBC: red blood cell; WHO: World Health Organization.

## Conflicts of interest

The authors declare that they have no competing interests.

## Authors' contributions

PG, ADW, SM, EB and JJ designed the study; PG, AS, JS, ADW and EB organized laboratory and clinical supervision in Tete; PG, AS, JS, HC and OJV actively participated in the laboratory work in Maputo and Antwerp; PG, AS, ADW, EB and JJ assessed and interpreted the results. All authors contributed to the discussion of the results and the redaction of the manuscript, they all approved the final manuscript.

## References

[B1] World Health Organization World malaria report 2010 2010 http://whqlibdoc.who.int/publications/2010/9789241564106_eng.pdf

[B2] Forney JR Magill AJ Wongsrichanalai C Sirichaisinthop J Bautista CT Heppner DG Miller RS Ockenhouse CF Gubanov A Shafer R DeWitt CC Quino-Ascurra HA Kester KE Kain KC Walsh DS Ballou WR Gasser RA Jr Malaria rapid diagnostic devices: performance characteristics of the ParaSight F device determined in a multisite field study J Clin Microbiol 2001 39 2884 2890 10.1128/JCM.39.8.2884-2890.2001 11474008 PMC88255

[B3] Marx A Pewsner D Egger M Nuesch R Bucher HC Genton B Hatz C Juni P Meta-analysis: accuracy of rapid tests for malaria in travelers returning from endemic areas Ann Intern Med 2005 142 836 846 15897534 10.7326/0003-4819-142-10-200505170-00009

[B4] Murray CK Gasser RA Magill AJ Miller RS Update on rapid diagnostic testing for malaria Clin Microbiol Rev 2008 21 97 110 10.1128/CMR.00035-07 18202438 PMC2223842

[B5] Ohrt C Obare P Nanakorn A Adhiambo C Awuondo K O'Meara WP Remich S Martin K Cook E Chretien JP Lucas C Osoga J McEvoy P Owaga ML Odera JS Ogutu B Establishing a malaria diagnostics centre of excellence in Kisumu, Kenya Malar J 2007 6 79 10.1186/1475-2875-6-79 17565676 PMC1933544

[B6] Pieroni P Mills CD Ohrt C Harrington MA Kain KC Comparison of the *Para*Sight™-F test and the ICT Malaria Pf™ test with the polymerase chain reaction for the diagnosis of *Plasmodium falciparum *malaria in travellers Trans R Soc Trop Med Hyg 1998 92 166 169 10.1016/S0035-9203(98)90730-1 9764322

[B7] Butch AW Dilution protocols for detection of hook effects/prozone phenomenon Clin Chem 2000 46 1719 1721 11017960

[B8] Heidelberger M Kendall FE A quantitative theory of the precipitin reaction: II. a study of an azoprotein-antibody system J Exp Med 1935 62 467 483 10.1084/jem.62.4.467 19870428 PMC2133297

[B9] Gillet P Mori M Van Esbroeck M Van Den Ende J Jacobs J Assessment of the prozone effect in malaria rapid diagnostic tests Malar J 2009 8 271 10.1186/1475-2875-8-271 19948018 PMC2789093

[B10] Mabunda S Casimiro S Quinto L Alonso P A country-wide malaria survey in Mozambique. I. *Plasmodium falciparum *infection in children in different epidemiological settings Malar J 2008 7 216 10.1186/1475-2875-7-216 18950486 PMC2579920

[B11] Mabunda S Aponte JJ Tiago A Alonso P A country-wide malaria survey in Mozambique. II. Malaria attributable proportion of fever and establishment of malaria case definition in children across different epidemiological settings Malar J 2009 8 74 10.1186/1475-2875-8-74 19383126 PMC2678146

[B12] Ministério da Saùde and Republica de Moçambique Manual de formação para o manejo de casos de malària 2009 Maputo

[B13] World Health Organization Guidelines for the treatment of malaria 2010 2 Geneva 25473692

[B14] Bruneel F Hocqueloux L Alberti C Wolff M Chevret S Bedos JP Durand R Le Bras J Regnier B Vachon F The clinical spectrum of severe imported falciparum malaria in the intensive care unit: report of 188 cases in adults Am J Respir Crit Care Med 2003 167 684 689 10.1164/rccm.200206-631OC 12411286

[B15] Bruneel F Tubach F Corne P Megarbane B Mira JP Peytel E Camus C Schortgen F Azoulay E Cohen Y Georges H Meybeck A Hyvernat H Trouillet JL Frenoy E Nicolet L Roy C Durand R Le Bras J Wolff M Severe imported falciparum malaria: a cohort study in 400 critically ill adults PLoS One 2010 5 e13236 10.1371/journal.pone.0013236 20949045 PMC2951913

[B16] World Health Organization Malaria rapid diagnostic test performance; Results of WHO product testing of malaria RDTs: Round 1 (2008) 2009 http://www.finddiagnostics.org/resource-centre/reports_brochures/malaria-diagnostics-report-2009.html21605401

[B17] World Health Organization Malaria Rapid diagnostic test performance; Results of WHO product testing of malaria RDTs: Round 2 (2009) 2010 http://www.finddiagnostics.org/resource-centre/reports_brochures/malaria-diagnostic-test-report-round2.html21605401

[B18] World Health Organization List of known commercially-available antigen-detecting malaria RDTs with adequate evidence of good manufacturing practice 2009 http://www.wpro.who.int/internet/resources.ashx/RDT/docs/MD_table34+(1)_totallistofISO131485criteria.pdf

[B19] Van der Palen M Gillet P Bottieau E Cnops L Van Esbroeck M Jacobs J Test characteristics of two rapid antigen detection tests (SD FK50 and SD FK60) for the diagnosis of malaria in returned travellers Malar J 2009 8 90 10.1186/1475-2875-8-90 19416497 PMC2688521

[B20] Batwala V Magnussen P Nuwaha F Are rapid diagnostic tests more accurate in diagnosis of plasmodium falciparum malaria compared to microscopy at rural health centres? Malar J 2010 9 349 10.1186/1475-2875-9-349 21126328 PMC3002380

[B21] Bjorkman A Martensson A Risks and benefits of targeted malaria treatment based on rapid diagnostic test results Clin Infect Dis 2010 51 512 514 10.1086/655689 20642355

[B22] Mawili-Mboumba DP Bouyou Akotet MK Ngoungou EB Kombila M Evaluation of rapid diagnostic tests for malaria case management in Gabon Diagn Microbiol Infect Dis 2010 66 162 168 10.1016/j.diagmicrobio.2009.09.011 19846265

[B23] Ochola LB Vounatsou P Smith T Mabaso ML Newton CR The reliability of diagnostic techniques in the diagnosis and management of malaria in the absence of a gold standard Lancet Infect Dis 2006 6 582 588 10.1016/S1473-3099(06)70579-5 16931409

[B24] World Food Programme TRMM precipitation data analysis 2011 http://reliefweb.int/sites/reliefweb.int/files/resources/42CE4923C3A7CEDA852 577430064DCAC-map.pdf

[B25] Makler MT Piper RC Rapid malaria tests: where do we go after 20 years? Am J Trop Med Hyg 2009 81 921 926 10.4269/ajtmh.2009.09-0202 19996417

[B26] Harvey SA Jennings L Chinyama M Masaninga F Mulholland K Bell DR Improving community health worker use of malaria rapid diagnostic tests in Zambia: package instructions, job aid and job aid-plus-training Malar J 2008 7 160 10.1186/1475-2875-7-160 18718028 PMC2547110

[B27] Mayxay M Newton PN Yeung S Pongvongsa T Phompida S Phetsouvanh R White NJ Short communication: An assessment of the use of malaria rapid tests by village health volunteers in rural Laos Trop Med Int Health 2004 9 325 329 10.1111/j.1365-3156.2004.01199.x 14996360

[B28] Rennie W Phetsouvanh R Lupisan S Vanisaveth V Hongvanthong B Phompida S Alday P Fulache M Lumagui R Jorgensen P Bell D Harvey S Minimising human error in malaria rapid diagnosis: clarity of written instructions and health worker performance Trans R Soc Trop Med Hyg 2007 101 9 18 10.1016/j.trstmh.2006.03.011 17049572

[B29] Risch L Bader M Huber AR [False negative quick malaria test] Schweiz Med Wochenschr 1999 129 1002 10431325

[B30] Gerstl S Dunkley S Mukhtar A De Smet M Baker S Maikere J Assessment of two malaria rapid diagnostic tests in children under five years of age, with follow-up of false-positive pLDH test results, in a hyperendemic falciparum malaria area, Sierra Leone Malar J 2010 9 28 10.1186/1475-2875-9-28 20092620 PMC2835716

[B31] Bisoffi Z Sirima SB Menten J Pattaro C Angheben A Gobbi F Tinto H Lodesani C Neya B Gobbo M Van Den Ende J Accuracy of a rapid diagnostic test on the diagnosis of malaria infection and of malaria-attributable fever during low and high transmission season in Burkina Faso Malar J 2010 9 192 10.1186/1475-2875-9-192 20609211 PMC2914059

[B32] Stow NW Torrens JK Walker J An assessment of the accuracy of clinical diagnosis, local microscopy and a rapid immunochromatographic card test in comparison with expert microscopy in the diagnosis of malaria in rural Kenya Trans R Soc Trop Med Hyg 1999 93 519 520 10.1016/S0035-9203(99)90359-0 10696409

[B33] Wiese L Bruun B Baek L Friis-Moller A Gahrn-Hansen B Hansen J Heltberg O Hojbjerg T Hornstrup MK Kvinesdal B Gomme G Kurtzhals JA Bedside diagnosis of imported malaria using the Binax Now malaria antigen detection test Scand J Infect Dis 2006 38 1063 1068 10.1080/00365540600818011 17148078

[B34] Chiodini PL Bowers K Jorgensen P Barnwell JW Grady KK Luchavez J Moody AH Cenizal A Bell D The heat stability of Plasmodium lactate dehydrogenase-based and histidine-rich protein 2-based malaria rapid diagnostic tests Trans R Soc Trop Med Hyg 2007 101 331 337 10.1016/j.trstmh.2006.09.007 17212967

[B35] Drakeley C Reyburn H Out with the old, in with the new: the utility of rapid diagnostic tests for malaria diagnosis in Africa Trans R Soc Trop Med Hyg 2009 103 333 337 10.1016/j.trstmh.2008.10.003 19019399

[B36] Fogg C Twesigye R Batwala V Piola P Nabasumba C Kiguli J Mutebi F Hook C Guillerm M Moody A Guthmann JP Assessment of three new parasite lactate dehydrogenase (pan-pLDH) tests for diagnosis of uncomplicated malaria Trans R Soc Trop Med Hyg 2008 102 25 31 10.1016/j.trstmh.2007.09.014 18031779

[B37] Bell D Peeling RW Evaluation of rapid diagnostic tests: malaria Nat Rev Microbiol 2006 4 S34 S38 10.1038/nrmicro1524 17034070

[B38] Olliaro P Management of fever and malaria - policy and practice Trop Med Int Health 2009 14 488 490 10.1111/j.1365-3156.2009.02272.x 19320873

[B39] D'Acremont V Lengeler C Mshinda H Mtasiwa D Tanner M Genton B Time to move from presumptive malaria treatment to laboratory-confirmed diagnosis and treatment in African children with fever PLoS Med 2009 6 e252 10.1371/journal.pmed.0050252 19127974 PMC2613421

[B40] Farcas GA Soeller R Zhong K Zahirieh A Kain KC Real-time polymerase chain reaction assay for the rapid detection and characterization of chloroquine-resistant *Plasmodium falciparum *malaria in returned travelers Clin Infect Dis 2006 42 622 627 10.1086/500134 16447106

[B41] Gillet P Mukadi P Vernelen K Van Esbroeck M Muyembe JJ Bruggeman C Jacobs J External quality assessment on the use of malaria rapid diagnostic tests in a non-endemic setting Malar J 2010 9 359 10.1186/1475-2875-9-359 21144034 PMC3019163

[B42] Msellem MI Martensson A Rotllant G Bhattarai A Stromberg J Kahigwa E Garcia M Petzold M Olumese P Ali A Bjorkman A Influence of rapid malaria diagnostic tests on treatment and health outcome in fever patients, Zanzibar: a crossover validation study PLoS Med 2009 6 e1000070 10.1371/journal.pmed.1000070 19399156 PMC2667629

[B43] World Health Organization Information note on interim selection criteria for procurement of malaria rapid diagnostic tests (RDTs) 2010 http://www.who.int/entity/malaria/diagnosis_treatment/diagnosis/infoRDTinterimcriteria.pdf

